# 

**Published:** 1860-09

**Authors:** 


### CONTENTS

OF THE

### Chicago Medical Journal for September, 1860.

ORIGINAL COMMUNICATIONS. PAGE.

ARTICLE 1.—Cancer of Mamma—Operation for. By T. Wilkins, M. D., Vandalia, Ill...... 505

ARTICLE 2.—Sympathetic Ophthalmitis, and Certain Forms of Chronic Iritis. By E. L. Holmes, M. D........ 508

SELECTIONS.

Mental Peculiarities and Mental Disorders of Childhood........ 513

Effects of Disease on the Teeth........ 519

Iodide of Potassium in Diseases of the Brain in Children........ 524

Observations on Revaccination........ 527

Extirpation of Glandula Parotidea........ 529

Extract from Lectures on Experimental Pathology and Operative Physiology........ 537

Ipecacuanha Instead of Tartar Emetic in Croup........ 543

Nourishment in Typhoid Fever........ 544

Sun Stroke........ 546

Bellevue Hospital........ 548

Chloroform in Obstetrics........ 550

Muriate of Ammonia in Nervous Cephalalgia........ 551

EDITORIAL.

Book and Pamphlet Notices........ 553 to 561

To Subscribers in Arrears........ 562

April and July Meetings of the Hancock County Medical Association........ 562

Stark County Medical Society........ 564

Rush Medical College........ 565

Diphtheria........ 566

Illinois State Medical Society........ 567

J. H. Reed & Co........ 567

